# Ethnic differences in the +405 and −460 vascular endothelial growth factor polymorphisms and peripheral neuropathy in patients with diabetes residing in a North London, community in the United Kingdom

**DOI:** 10.1186/s12883-017-0905-3

**Published:** 2017-06-29

**Authors:** Karima Zitouni, Lorna Tinworth, Kenneth Anthony Earle

**Affiliations:** 1 Department of Cellular and Molecular Medicine, St. Georges University of London, London, UK; 2 Department of Biomedical Sciences, University of Westminster, London, UK; 3 St Georges University Hospitals NHS Foundation Trust, Thomas Addison Diabetes Unit, London, UK

**Keywords:** Diabetes, Peripheral neuropathy, Ethnicity, VEGF, Gene polymorphism, Pyrosequencing, Microvascularization

## Abstract

**Background:**

There are marked ethnic differences in the susceptibility to the long-term diabetic vascular complications including sensory neuropathy. The vascular endothelial growth factor (VEGF) +405 (C/G) and −460 (T/C) polymorphisms are associated with retinopathy and possibly with nephropathy, however no information is available on their relationship with peripheral neuropathy. Therefore, we examined the prevalence of these VEGF genotypes in a multi-ethnic cohort of patients with diabetes and their relationship with evident peripheral diabetic neuropathy.

**Methods:**

In the current investigation, we studied 313 patients with diabetes mellitus of African-Caribbean, Indo-Asian and Caucasian ethnic origin residing in an inner-city community in London, United Kingdom attending a single secondary care centre. Genotyping was performed for the VEGF +405 and VEGF −460 polymorphisms using a pyrosequencing technique.

**Results:**

Forty-nine patients (15.6%) had clinical evidence of peripheral neuropathy. Compared to Caucasian patients, African-Caribbean and Indo-Asian patients had lower incidence of neuropathy (24.6%, 14.28%, 6.7%, respectively; *P* = 0.04). The frequency of the VEGF +405 GG genotype was more common in Indo-Asian patients compared to African-Caribbean and Caucasian patients (67.5%, 45.3%, 38.4%, respectively; *p* ≤ 0.02). The G allele was more common in patients with type 2 diabetes of Indo-Asian origin compared to African-Caribbean and Caucasian origin (*p* ≤ 0.02). There was no difference between the ethnic groups in VEGF −460 genotypes. The distributions of the VEGF +405 and VEGF −460 genotypes were similar between the diabetic patients with and without neuropathy.

**Conclusions:**

In this cohort of patients, VEGF +405 and VEGF −460 polymorphisms were not associated with evident diabetic peripheral neuropathy, however an association was found between VEGF +405 genotypes and Indo-Asian which might have relevance to their lower rates of ulceration and amputation. This finding highlights the need for further investigation of any possible relationship between VEGF genotype, circulating VEGF concentrations and differential vulnerability to peripheral neuropathy amongst diabetic patients of different ethnic backgrounds.

## Background

Peripheral neuropathy affects up to 50% of patients with diabetes [1]. It is a major risk factor that makes diabetes responsible for 50–75% of non-traumatic amputations in the world [2, 3]. The pathogenesis of diabetic neuropathy is poorly understood, but is likely to be multifactorial, a result of genetic susceptibility, environmental and lifestyle factors [4, 5].

There is increasing evidence that the prevalence of peripheral neuropathy as a complication of diabetes varies according to race/ethnicity. The North American and European studies of multi-ethnic groups have found a greater prevalence of neuropathy occurring in Caucasian patients compared to other ethnic groups [6]. Population based studies in the United Kingdom (UK) have reported lower rates of peripheral neuropathy in patients with type 2 diabetes of Indo-Asian and African-Caribbean origin compared with those of Caucasian origin [7–9]. Studies showing evidence of increased skin microvascularization in Indo-Asian subjects has been considered a cause for the lower rates of neuropathy compared with Caucasian subjects [10]. These findings were independent of glycaemic control and traditional risk factors for vascular disease, suggesting that genetic, or some other, as yet unidentified protective factor(s) may be relevant to these observations.

Strict metabolic control of hyperglycaemia is essential in preventing the onset and progression of diabetic neuropathy [2, 3]. Hyperglycaemia disrupts sensory nerve function through several mechanisms including, local hypoxia, activation of protein kinase C, formation of advanced glycation end products, oxidative stress and pro-inflammatory changes [4, 5]. A counter-response to these metabolic perturbations is the stimulation of gene expression of the angiogenic cytokine, Vascular Endothelial Growth Factor (VEGF) which attempts to alleviate hypoxia and improve nutrient supply.

A positive correlation has been reported between stimulated peripheral blood mononuclear cell VEGF protein production and the +405 GC VEGF gene polymorphism [11]. The highest VEGF protein production was noted in individuals who were +405 GG homozygous, while the lowest production was seen in subjects who were +405CC. In patients with diabetes, several authors have shown associations between the VEGF +405 and VEGF −460 genotypes with microvascular and macrovascular complications [12–16]. However, no study has yet investigated the relationship of these polymorphisms and the vulnerability to diabetic neuropathy.

Therefore, the aim of this study is to determine the distribution of VEGF +405 and VEGF −460 gene (Genebank numbers rs2010963 and *rs833061*) polymorphisms in Indo-Asian, African-Caribbean, and Caucasian patients with diabetes and examine the relationship with clinical evidence of diabetic peripheral neuropathy.

## Methods

### Patients

The study was conducted at a secondary care centre in North London, UK, serving an inner-city community of 154,000 adults. Consecutive adult, attendees to the diabetes unit of Caucasian, Indo-Asian or African-Caribbean ethnicity were invited to take part in the study. Patients were considered to be of African-Caribbean or Indo-Asian ethnicity if both parents were native to either African or Caribbean countries or India, Pakistan, Bangladesh or Sri Lanka respectively. The parents of white, Caucasian patients were native of Western European or Mediterranean countries. A diagnosis of type 2 diabetes was based on an absence of ketosis or need of insulin within 1 year of diagnosis. Type 1 diabetes was confirmed in each subject on the basis of the diagnosis being made at less than 35 years of age with a history of ketosis at presentation and continuous use of subcutaneous insulin within 1 year from diagnosis. Patients with history of malignancy were excluded.

Patients were diagnosed as having peripheral sensory neuropathy if they had diminished sensation to light touch with the 10 g monofilament or to pinprick. Body mass index (BMI) was calculated from weight in kilograms divided by height in meters squared. Sitting blood pressure was measured after 10 min rest using a validated automated machine (OMRON 705HEM CP; OMRON Healthcare, West Sussex, U.K.) using an appropriate cuff size.

Fasting venous blood was taken from an antecubital vein. Glycosylated Haemoglobin A1c (HbA1c) was measured by a high-performance liquid chromatography system (Menarini 8140; Menarini Diagnostics, Wokingham, U.K.). Total cholesterol, HDL cholesterol and total triglycerides were estimated using enzymatic methods (Boehringer-Mannheim, Mannheim, Germany). The samples were coded in order to blind the laboratory staff to the patients’ sex, age, racial origin, diabetes, and comorbidity status.

The study was approved by the ethics committee of the Whittington Hospital National Health Service Trust. Written, informed consent was provided by all patients entering the study.

### DNA extraction

A modification of the method described by Miller et al. [17] was used. Briefly, 1 mL of the blood collected in sodium citrate and stored at −80 °C was re-suspended in 2 mL of erythrocyte lysis solution (1.44 mM ammonium chloride, 1 mM sodium bicarbonate) and the suspension centrifuged at 15,000×*g* for 2 min. The supernatant was discarded, and the pellet was re-suspended in 0.64 mL nuclear lysis buffer [(10 mM Tris-HCl (pH = 8.2), 0.4 M sodium chloride, 2 mM di-sodium EDTA (pH = 8.0)], 0.18 mL sodium chloride, and chloroform. The samples were shaken vigorously and centrifuged at 10, 000×g for 3 min. The supernatant containing the DNA was transferred into new eppendorf tubes and 1 mL of 100% ethanol was added and the tubes were inverted repeatedly. The samples were centrifuged for 2 min at 10, 000×g. The supernatant was discarded and the pellet, containing DNA was dissolved in %70 ethanol and the samples were centrifuged at 10, 000×g for 2 min. The upper layer was then removed and polymerase chain reaction (PCR) grade water was added. The concentration and purity of DNA was measured using a NanoDrop spectrophotometer (NanoDrop Technologies, Wilmington, DE).

### Polymerase chain reaction

Polymerase chain reaction procedure was performed in a total volume of 25 μL reaction mixture that contained “Red Hot” Taq DNA polymerase enzyme (0.5 U), genomic DNA (250 ng), 20 pmol of each primer set (sequences shown in Table 1) and Mg2+ (1.5 mM).

**Table 1:** Primer sequences

	VEGF + 405	VEGF-460
Forward primers sequence (5′-3′)	GAGAAGTCGAGGAAGAGAGAGACG	AATGGAGCGAGCAGCGTCTT
Reverse primer sequence (5′-3′)	CCCCAAAAGCAGGTCAC	CGTTCCCTCTTTGCTAGGAATAT
Sequencing primers(5′-3′)	GTGCGAGCAGCGAAA	TGCGTGTGGGGTTGA

All PCR mixes were amplified with the same touch down program: Denature at 95 °C for 5 min followed by 35 cycles of [denaturation (95 °C for 1 min), annealing (55 °C for 1 min) and extension (72 °C for 1 min)] and a final step of extension at 72 °C for 10 min.

### Pyrosequencing

PCR product (10 μL) was immobilised onto sepharose streptavidin-coated beads (Amersham Bioscience, Amersham, UK) suspended in binding buffer (10 mM Tris-HCl, 2 M NaCl, 1 mM EDTA, 0.1% Tween 20, pH 7.6) and water to a total volume of 70 μL, at room temperature (21 °C) for 5 min. Single-stranded (ss) DNA was obtained by washing the immobilised PCR product for 5 s successively in 70% ethanol, 0.2 M NaOH and washing buffer (10 mM Tris-acetate) followed by 12 μL annealing buffer (20 mM Tris-Acetate, 5 mM magnesium acetate, pH 7.6) containing 3.0 μM sequencing primer (Table 1) in a 96-well PSQ HS plate. The primer was annealed to the ssDNA template by heating the plate at 80 °C for 2 min, then allowing it to cool at room temperature (21 °C). Pyrosequencing analysis was carried out using the PSQ96 HS 96A instrument and the SNP Reagent kit containing dNTPs, enzyme and substrate mixtures according to manufacturer’s protocols (Pyrosequencing AB, Uppsala, Sweden).

### Statistics

Data were analysed using SPSS 19 (SPSS, Chicago, IL, USA). Biochemical and clinical data are expressed as mean ± standard deviation. Continuous data were analysed with parametric or non-parametric tests according to the distribution. The chi-square test was used to compare the categorical data, genotyping and allelic distributions between patients with neuropathy and without, genotyping and allelic distributions between the ethnic groups. Logistic regression analysis was performed with neuropathy as the dependent variable to analyse the effect of ethnic group, VEGF genotype and glycated haemoglobin. A value of *p* ≤ 0.05 was accepted as significant.

## Results

**Fig. 1:**
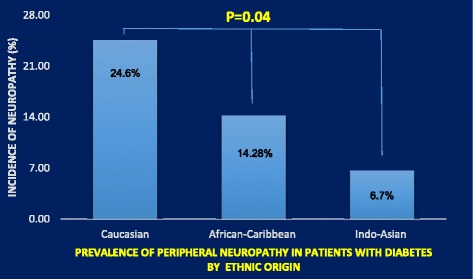
Prevalence of peripheral neuropathy in patients with diabetes from Caucasian, African Caribbean and Indo-Asian origin)

Three hundred and thirteen diabetic patients were included in this study, of which forty nine (15.6%) patients had peripheral neuropathy (Table 2). The prevalence of peripheral neuropathy was highest in the Caucasian group (24.6%) compared to the African-Caribbean (14.28%) and the Indo-Asian ethnic groups (6.7%) (*P* = 0.04) (Fig. 1). Patients from the Caucasian origin had more type 1 diabetes per comparison to African-Caribbean and Indo-Asian patients, while the latter had less proportion of smokers per comparison to the other ethnic groups (Table 3).

**Table 2:** Demographic and clinical characteristics of diabetic patients with and without peripheral neuropathy

DEMOGRAPHIC/CLINICAL parameters	With Neuropathy (*n* = 49)	Without neuropathy (*n* = 264)
Age (years)	67.48 ± 8.18**	60.60 ± 11.29
Diabetes(Type1/Type2)%	8.6/91.4	12.9/87.1
Duration of diabetes (years)	22.16 ± 9.45**	15.73 ± 8.01
Gender (male/female)%	65.6/34.4*	46.6/52.9
Smoking history (yes/No) %	78.1/21.9	73.8/26.2
Caucasian/African-Caribbean/Indo-Asian (%)	77.55/18.36/4.08*	58.50/27.35/14.15
Systolic blood pressure (mmHg)	143.04 ± 20.77	144.16 ± 21.68
Diastolic blood pressure (mmHg)	77.36 ± 10.14	81.14 ± 11.24
Hypertensive (%)	65.3	65.1
Total Cholesterol (mmol/L)	4.5 ± 1.4	4.67 ± 1.19
HDL-Cholesterol (mmol/L)	1.19 ± 0.29	1.43 ± 0.41
Triglycerides(mmol/L)	2.33 ± 1.99	1.99 ± 1.4
BMI (Kg/m2)	31.98 ± 4.86	31.03 ± 6.57
HbA1c % (mmol/mol)	8.07 ± 1.52 (65 ± 5.0)	8.00 ± 1.59 (64 ± 11.0)

Data expressed as mean ± SD

*p ≤ 0.05

**p ≤ 0.001

**Table 3:** Demographic and clinical characteristics of Indo-Asian, African-Caribbean and Caucasian patients

DEMOGRAPHIC/CLINICAL parameters	Indo-Asian	African-Caribbean	Caucasian
Age (years)	59.47 ± 10.62	56.49 ± 12.06	56.90 ± 15.78
Diabetes(Type1/Type2)%	6.06/93.94	3.08/96.92	21.47/78.53*
Duration of diabetes (years)	17.34 ± 7.26	15.42 ± 8.65	16.66 ± 10.27
Gender (male/female)%	61.8/38.2	43.8/56.2	57.5/42.5
Smoking history (yes/No) %	51.9/48.1	73.7/26.7	76.1 /23.9*
Systolic blood pressure (mmHg)	147.82 ± 24.42	145.62 ± 21.33	140.33 ± 23.02
Diastolic blood pressure(mmHg)	82.33 ± 10.70	83.35 ± 12.62	79.11 ± 11.09
Total Cholesterol (mmol/L)	4.73 ± 0.91	4.59 ± 1.18	4.98 ± 0.92
HDL-Cholesterol (mmol/L)	1.3 ± 0.64	1.42 ± 0.36	1.35 ± 0.36
Triglycerides (mmol/L)	2.22 ± 1.12	1.67 ± 0.94	2.140 ± 1.13
Height (m)	1.62 ± 0.10	1.65 ± 0.09	1.66 ± 0.11
BMI (Kg/m2)	27.37 ± 4.57	30.73 ± 6.43	31.66 ± 7.37
HbA1c %	7.5 ± 1.24	8.33 ± 1.78	7.9 ± 1.62

Data expressed as mean ± SD

*p ≤ 0.005

The patients with peripheral neuropathy had significantly longer duration of diabetes, were older, taller and were more likely to be men. There were no significant differences in HbA1c as a measure of glycemic control, total cholesterol, HDL-cholesterol, and triglyceride levels between the two groups of patients. The groups had similar BMI, levels of systolic and diastolic blood pressure, treatment for hypertension and smoking history (Table 2).

### VEGF +405 and −460 polymorphisms and ethnic group

There was a marked disparity in the distribution of the VEGF +405 genotypes between the Indo-Asian, African-Caribbean, and Caucasian patients (Table 4). The VEGF +405 GG was more common in Indo-Asians (67.5%) compared to African-Caribbean (45.3%) and Caucasian patients (38.4%) (*p* ≤ 0.05 and *p* ≤ 0.005, respectively). The G allele was more common while the C allele was less common in the Indo-Asian group per comparison to the African-Caribbean and Caucasian ethnic groups (*p* ≤ 0.02 and *p* ≤ 0.005, respectively). There were no interethnic differences in VEGF −460 genotypes (data not shown).

**Table 4:** VEGF +405 genotypes in type 2 diabetes patients from Indo-Asian, African Caribbean and Caucasian origin

VEGF + 405 genotypes	Indo-Asian	African-Caribbean	Caucasian
CG (%)	13 (32.5)^a, b^	36 (48.0)	79 (57.25)
CC (%)	0 (0)	5 (6.7)	6 (4.35)
GG (%)	27 (67.5)^a, b^	34 (45.3)	53 (38.4)
C (%)	13 (16.25)^c, d^	46 (30.67)	91 (32.97)
G (%)	67 (83.75)^c, d^	104 (69.33)	185 (67.03)

^a^Compared to African Caribbean (χ^2^ = 6.5541, *p* ≤ 0.05)

^b^Compared to Caucasian (χ^2^ = 11.2541, *p* ≤ 0.005)

^c^Compared to African Caribbean (χ^2^ = 5.6858, *p* ≤ 0.02)

^d^Compared to Caucasian (χ^2^ = 8.3857, *p* ≤ 0.005)

### VEGF +405 and VEGF −460 gene polymorphisms and peripheral neuropathy

Genotype frequencies for the +405 C⁄G at the exon 1 of VEGF gene between patients with peripheral neuropathy and without peripheral neuropathy are shown in Table 5.

**Table 5:** Genotype and Allele frequencies of the VEGF + 405 gene polymorphisms in patients with and without neuropathy

VEGF + 405 Polymorphism	With Neuropathy	Without neuropathy
1) Whole population Genotypes	*N* = 35	*N* = 264
CG (%)	18 (51.4)	134 (50.8)
GG (%)	16 (45.7)	119 (45.1)
CC (%)	1 (2.9)	11 (4.2)
C (%)	21 (29.58)	156 (29.55)
G (%)	50 (70.42)	372 (70.45)
2) Type 2 diabetes Genotypes	*N* = 32	*N* = 221
CC (%)	1 (3.1)	10 (4.5)
CG (%)	18 (56.3)	110 (49.8)
GG (%)	13 (40.6)	101 (45.6)
C (%)	20 (31.25)	130 (29.41)
G (%)	44 (68.75)	312 (70.59)

In the analysis of patients with type 2 diabetes only cohort (*n* = 253), there was no statistically significant difference in the VEGF +405 genotype distribution nor the C and G Allele frequencies (*p* = 0.76) between the patients with and without peripheral neuropathy (Table 5).

There was no statistically significant difference between C and T alleles (*p* = 0.73) and genotype frequency of the -460C ⁄T. The analyses also failed to find an association between G, C alleles (*p* = 0.99) or +405C ⁄G VEGF polymorphism between patients with and without peripheral neuropathy (Table 5). In the logistic regression analysis model with peripheral neuropathy as the dependent variable we tested the association between the VEGF +405 polymorphism and the quality of diabetes control HbA1c. The odds ratio (95% confidence interval) was 0.67 (0.47-0.97) for patients of non-Caucasian origin (*p* = 0.035) and 1.25 (1.04 – 1.50) for HbA1c (*p* = 0.015).

## Discussion

We found that the distribution of the genetic polymorphisms of VEGF +405 and VEGF-460 varied with ethnic origin, but differences were not found to be associated with the presence of peripheral neuropathy in this cohort of patients with diabetes. Patients of non-Caucasian origin were 33% less likely to have peripheral neuropathy compared to Caucasian patients after correcting for HbA1c.

In recent years, the association of VEGF and diabetic neuropathy has attracted much attention. Vascular endothelial growth factor is an angiogenic cytokine that can be induced by hypoxia and increases capillary permeability, promote the synthesis of extracellular matrix and promote the proliferation and hypertrophy of endothelial cells [18]. However, VEGF can have deleterious effects on the basement membrane of vessels, increasing endoneurial vascular permeability, leading to nerve fibre ischemia and hypoxia, and thus contributing to the occurrence of neuropathy [19, 20].

In animal models of diabetes and neuropathy, increased VEGF staining has been reported in neuronal cell bodies in dorsal root ganglia, axons and in Schwann cells of peripheral nerves, also, higher circulating levels of the cytokine have been reported in comparison to healthy animals [21, 22]. Treatment with plasmid DNA encoding for VEGF was shown to improve sensory behavioural features and sciatic nerve blood flow [20]. Clinical studies using the intramuscular plasmid VEGF gene transfer have been shown to improve neuropathic symptoms in patients with diabetes [23].

The VEGF +405 and VEGF −460 gene polymorphisms were not shown to be associated with peripheral neuropathy in our patients. However whether VEGF accounts for the differential vulnerability to neuropathy amongst patients of different ethnic background remains to be determined. In our study, the Indo-Asian ethnic group with the lowest prevalence of neuropathy had the highest frequency of the +405 GG VEGF genotype, which has been shown to be associated with raised plasma VEGF levels [11]. It is reported that patients with type 2 diabetes of Indo-Asian and African-Caribbean origin are less likely to develop neuropathy and foot ulceration compared with those of Caucasian origin [7–10]. In our cohort though we found a near 3-fold greater prevalence of clinically evident peripheral neuropathy in the Caucasian patient group compared with the non-Caucasian group. The relatively high prevalence of peripheral neuropathy in this cohort is in part due to these patients being attendees of a secondary care centre and there are differences in methodologies reported in different centres for case finding. In contrast to other studies we used the 10 g monofilament in consecutive patients, whereas other studies used the neurothesiometer and biothesiometer in selected patients [8, 10, 24]. Patients of non-Caucasian background are generally poorly represented in research studies in the UK and the lack of standardisation in the methodologies to diagnose neuropathy may have affected the reported prevalence [25]. However, if the prevalence of neuropathy is similar between these ethnic groups there would have been clear ethnic similarities in the rates of penetrative ulceration and limb amputation. Insensitivity to the 10 g monofilament increases the 2 year risk of ulceration nearly 3-fold [26]. However, the rates of lower limb amputation amongst patients with diabetes of Indo-Asian compared to Caucasian origin are significantly lower in the UK [27].

In this cross-sectional study, the VEGF +405 polymorphism distribution was similar between patients of African-Caribbean and Caucasian origin and the frequencies of the G and C alleles were similar between the ethnic groups. These findings are in line with the only two other studies that looked at VEGF +405 polymorphism and ethnicity [28, 29]. Moreover, the frequencies found for the VEGF +405G Allele in this cohort of patients of Caucasian and African-Caribbean origin (67% and 69%, respectively) are similar to those reported in American whites and black individuals without diabetes and vascular disease [28] and in white and black Brazilians [29]. These findings suggest that VEGF +405 genotype distribution and allele frequencies are similar across ethnic groups when comparing Caucasian and African-Caribbean cohorts in the presence or absence of diabetes.

Our study found that VEGF +405 GG genotype was more common in patients of Indo-Asian origin in comparison to African-Caribbean and Caucasian patients. In addition, the frequency of the VEGF +405G allele was higher in the Indo-Asian patients (83.75%) per comparison to African-Caribbean and Caucasian patients (67% and 69%, respectively). Previous research demonstrated that Lipopolysaccharide stimulated peripheral blood mononuclear cell VEGF protein production was highest among individuals who were VEGF +405GG [11] and high expression of VEGF was protective for ischemic neuronal damage [30, 31]. It can be postulated that VEGF +405GG has a protective role against neuropathy and may explain why patients of Indo-Asian origin, should they develop ulceration may have better rates of healing, and therefore lower rates of amputation [26]. Furthermore, the VEGF −460 polymorphism, which has not been correlated to plasma VEGF concentration [11] was not different between the ethnic groups.

Although, this study did not measure serum VEGF concentration, elevated serum VEGF levels have been reported in healthy subjects with the VEGF +405CC genotype and not the VEGF +405 GG [12]. Lambrechts and colleagues [32] demonstrated that production of large-VEGF isoform with the VEGF +405G allele in cell extracts was 20% lower in comparison to VEGF +405C allele. It has also been shown that haplotypes of VEGF -2578A/−1154A/+405G and -2578A/−1154G/+405G in the VEGF promoter/leader lowered circulating VEGF levels in vivo and reduced VEGF gene transcription [32]. Similar results were reported in cultured human myoblasts that analysed the effects of the VEGF −2578/−1154/+405 promoter region haplotype on VEGF gene expression [28]. Moreover, ethnic differences in VEGF haplotypes -2578C/−1154G/+405G were reported to be more common in Black than White Brazilian individuals even though these groups had similar profile in VEGF + 405 genotype [29]. Collectively, these studies and our study findings suggest that ethnic differences in various VEGF polymorphisms exist, and the possible association between these VEGF genotypes including VEGF +405 might influence circulating VEGF concentrations.

The prevalence of peripheral neuropathy in this real-world selected cohort is in agreement with previous studies with regards to the vulnerability of Caucasian patients to peripheral neuropathy in comparison to other ethnic groups [7, 8, 24]. Glycaemic control is the major factor in the development of diabetic neuropathy [5, 24, 33]. In the current study, there were no differences in HbA1c in the patients with, compared to those without neuropathy. Furthermore, in correcting for glycaemic control non-Caucasian ethnic group appears to be a protective factor for the development of neuropathy.

A number of studies in small groups of patients have identified an association between diabetic neuropathy and VEGF gene polymorphisms, but not the propensity to develop diabetic neuropathy or the healing process. The VEGF gene SNP at −7*C/T was associated with diabetic neuropathy in type 1 diabetic patients of British Caucasian origin [34]. Another VEGF polymorphism that was associated with diabetic neuropathy is VEGF 936 C/T in type 2 diabetic Chinese patients [35]. The latest VEGF polymorphism that was reported to be associated with neuropathy is the Insertion/deletion VEGF gene that was studied in type 2 diabetes population [36].

## Conclusions

The present study is the first to examine the distribution of VEGF +405 and VEGF −460 genotypes in African-Caribbean, Indo-Asian and Caucasian patients with diabetes and the association of these genotypes with peripheral neuropathy. Although there was a difference in the VEGF + 405 polymorphism and the frequencies of G and C alleles between the ethnic groups, this study did not find any association with diabetic neuropathy regardless of diabetes type or ethnic origin. The results may suggest that neither VEGF +405 nor VEGF −460 polymorphisms are involved with diabetic peripheral neuropathy. However the complexity of VEGF gene expression and the small sample size of the enrolled population limits our conclusions. These findings support a hypothesis that the VEGF +405 genotype may confer protection from developing peripheral neuropathy and therefore the risk of developing foot ulceration and lower limb amputation in patients of non-Caucasian origin in the UK. This study results reiterate the need for further research into the possible relationship between VEGF genotypes, circulating VEGF concentrations and differential vulnerability to peripheral neuropathy in patients with diabetes of different ethnic backgrounds.

## Abbreviations

PCR: Polymerase chain reaction
VEGF: Vascular Endothelial Growth Factor
